# Carboxylated nanocellulose from quinoa husk for enhanced protease immobilization and stability of protease in biotechnological applications

**DOI:** 10.1038/s41598-024-77292-y

**Published:** 2025-01-02

**Authors:** Shohreh Ariaeenejad, Elaheh Motamedi

**Affiliations:** 1 Department of Systems and Synthetic Biology, Agricultural Biotechnology Research Institute of Iran (ABRII), Agricultural Research Education and Extension Organization (AREEO), Karaj, Iran; 2 Department of Nanotechnology, Agricultural Biotechnology Research Institute of Iran (ABRII), Agricultural Research Education and Extension Organization (AREEO), P. O. Box: 31535-1897, Karaj, Iran

**Keywords:** Carboxylated Nanocellulose, PersiProtease1, Immobilization, Thermal Stability, Recyclability., Biotechnology, Chemistry, Nanoscience and technology

## Abstract

Herein, an efficient and feasible approach was developed to oxidize low-cost agricultural waste (quinoa husk, QS) for the synthesis of carboxylated nanocellulose (CNC). The as-prepared rod-like CNCs (average diameter of 10 nm and length of 103 nm) with a high specific surface area (173 m^2^/g) were utilized for the immobilization of a model protease enzyme (PersiProtease1) either physically or via covalent attachment. For chemical immobilization, CNCs were firstly functionalized with N, N′-dicyclohexylcarbodiimide (DCC) to provide DCNCs nanocarrier which could covalently bond to enzyme trough nucleophilic substitution reaction and formation of the amide bond between DCNCs and enzyme. The immobilization efficiency, activity, stability, kinetic parameters, and reusability of covalently attached and physically immobilized PersiProtease1 were similar to those of the free enzyme. Enzyme immobilization resulted in higher thermal stability of the enzyme at elevated temperatures (> 80 °C), and the covalently immobilized enzyme displayed higher reusability than its physically immobilized form (56% vs. 37% activity, after 15 consecutive cycles), which would be rooted in a more tightly attached and less leached enzyme in the case of PersiProtease1/DCNCs. This study demonstrates the significance of using agricultural by-products and the enhanced performance and stability of immobilized proteases.

## Introduction

In recent years, interest in enzymatic biocatalysts has significantly increased because of their vital roles in numerous biological processes^1,2^. Their appeal stems from their ability to provide an eco-friendly framework for exploring reactions under mild conditions owing to their different specificities and selectivity^3^. However, the deployment of enzymes often faces challenges of diminished stability and activity, especially in the face of harsh operational parameters, such as extreme pH, temperature fluctuations, or the presence of antagonistic chemicals, all of which culminate in escalated commercial production expenses^4^. Immobilization techniques have emerged as leading strategies for enhancing enzyme stability and usability for wider applications^5–8^. Regarding advanced nanotechnology, different nanostructured materials can effectively be used for the immobilization of enzymes because of their advantages, such as large surface area, minimum mass transfer resistance, and high enzyme loading^9,10^. Nevertheless, selection of an appropriate nanocarrier is essential for the successful immobilization of enzymes. Recently, cellulosic-based nanocarriers (CNs), as innovative, biocompatible, affordable, and feasible supports for enzymes have received special interest for improving the efficiency, storage/operational stability, and reusability of enzymes^11^. The presence of hydroxyl functional groups and negative surface charges, along with viable chemical modifications on the surface of CNs, offer an effective enzyme immobilization opportunity via both physical and covalent conjugation strategies^12–14^. Moreover, among different biocatalysts, proteases are vital enzymes that are widely used in industrial operations^15^. Proteases are hydrolytic enzymes that hydrolyze large protein molecules into short peptides and amino acids^16^. These enzymes account for approximately 60% of the global enzyme market and have a broad range of applications in detergents, food beverages, textiles, medicine, pulp, and paper industries^17,18^. Currently, several methods are used for protease production. To ensure the probability of robust enzyme identification, metagenomics has emerged to overcome the limitations of culture-dependent methods^19^. Additionally, various potent enzymes have been identified through in-silico screening of metagenomic data and have been used in multiple industrial applications^20–22^. Recently, to gain higher enzyme functionality, a variety of metagenome-derived enzymes have been successfully immobilized on materials at the nanoscale size and utilized for various applications^10,12,14,23^. Similarly, several nanomaterials have been used to immobilize proteases. In an earlier study, protease was immobilized on 24 commercial carriers through five different immobilization strategies (physical adsorption, ionic binding, covalent binding, affinity binding, and entrapment), and the results confirmed an improvement in the stability of the immobilized enzymes toward metal ions and detergents and changes in the kinetic constants^24^. Likewise, applying physical adsorption, ionic binding, and covalent binding methods of immobilization improved the reusability and, pH, thermal, and storage stability of the protease^25,26^. In addition, a previous study reported the applicability of protease immobilized on zinc oxide nanoparticles to enhance the efficiency of the enzyme in leather processing^27^. In another study, the immobilization of proteases on chitosan enhanced the catalytic activity and stability of the enzymes^28,29^. Studies have indicated enhanced enzymatic performance of proteases by immobilization on carriers, such as glutaraldehyde-activated graphene oxide nanosheets^26^, poly(vinylimidazole)/clay hydrogel^30^, and agarose beads^31^.

Similar to other enzymes, protease applications would be widened/improved through immobilization as long as some limiting factors related to its specific application are taken into account. For instance, the successful application of an immobilized protease has been reported in the cheese-making industry via conducting hydrolysis at coagulation temperature for the prevention of immobilized enzyme trapping^32^. The immobilization carrier should be tailored according to the enzyme application, for example, porous carriers would be proper in the synthesis of peptides where modified small substrates are used, and they could protect the enzyme from the reaction medium which causes its inactivation by proteolysis, and interactions with interfaces (gas bubbles or drops of immiscible solvents)^33^. Nevertheless, when enzymes are used in processes with large and rigid substrates, porous carriers generate some problems because the pores of the carrier should be large enough to enable the entry of the substrate, not only of the enzyme^33^. In cases, where the proteases should be utilized under harsh conditions such as hydrolysis aggregated enzymes that are resolubilized in chaotropic agents, the need for a stable enzyme that could maintain its activity under concentrations of urea or guanidine for resolubilized aggregates is vital and very stable enzymes which immobilized by multipoint covalent bonds to their carriers are required^33^.

The current study aimed to synthesize proficient CNs for the effective immobilization of a metagenome-derived protease identified and characterized in our earlier study, PersiProtease1^34^. Carboxylated nanocellulose (CNC) obtained from agricultural waste can serve as a robust nanocarrier^35^. To achieve this, CNC was first synthesized from (quinoa husk, QS) via the reaction of sodium hydroxide-pretreated QS pulp with potassium permanganate (KMnO_4_) and oxalic acid (OA) reagents. Then, the CNCs were utilized for the immobilization of PersiProtease1 either physically or through chemical conjugation. It is hypothesized that covalent conjugation of PersiProtease1 to CNCs may offer higher stability/activity of the enzyme than its physically immobilized form. Physical immobilization was carried out through electrostatic attraction between the nanocarrier and enzyme. In contrast, the carboxylic acid groups on the CNCs were activated with N, N′-dicyclohexylcarbodiimide (DCC) to synthesize DCNCs carriers for covalent enzyme conjugation via amide bond formation between the carrier and PersiProtease1. The physically and covalently immobilized enzymes on CNCs were coded as PersiProtease1/CNCs and PersiProtease1/DCNCs, respectively, and their activities and stabilities were compared and contrasted under different conditions. This research highlights the kinetic and stability analyses and establishes a robust basis for the potential use of synthesized CNCs in biocatalysis and various biotechnological applications. Thorough examination of both covalently and physically immobilized PersiProtease1, in terms of immobilization efficiency, activity, stability, kinetic parameters, and reusability, offers detailed insight into the benefits of covalent immobilization compared to physical immobilization, which is essential for advancing further studies and industrial implementations.

## Materials and methods

### Chemicals

For preparation of nanocarriers, quinoa husk was provided by Seed and Plant Research Improvement Institute (SPII), Karaj, Iran. Potassium permanganate (KMnO_4_), oxalic acid (OA), sodium hydroxide (NaOH), dimethylformamide (DMF), and N, N′-dicyclohexylcarbodiimide (DCC) were obtained from Sigma–Aldrich. For characterization of the free and immobilized enzymes, casein, Na_2_CO_3_, Trichloroacetic acid (TCA) and Folin from Sigma–Aldrich were used. All the solutions were prepared in analytical grade and deionized water and buffers with appropriate pH were utilized.

### Synthesis of nanocarriers

For preparation of CNCs, the raw QS was grounded to the fine powder using a blender and then 2.5 g of it was dispersed in 100 ml NaOH solution (4% w/v) and stirred for 24 h, at room temperature. Next, the NaOH-treated QS precipitates were washed with distilled water, collected using centrifugation, and dried at 70 °C. In the next step, 1 g of the dried treated QS was dispersed in 50 mL distilled water contained sulfuric acid (1%), and consequently 2 g of KMnO_4_, and 1 g of OA were added to the flask gradually and stirred at 50 ºC, for 4 h. The precipitates were collected and washed with distilled water to increase the pH of the suspension to 5, and the resultant was dried at 70 °C.

For preparation of DCNCs carrier, 300 mg of dried CNCs powder was added to 100 mL of DMF and suspended using an ultrasonic bath. Then DCC (150 mg) was added to CCNCs suspension and stirred overnight, at room temperature. The precipitates were collected, washed, and dried at 70 °C.

### Characterization of nanocarriers

Field emission scanning electron microscopy (FESEM, TESCAN MIRA II microscope; 20 kV), transmission electron microscopy (TEM, Philips EM 208 S, 100 kV), Fourier-transform infrared spectroscopy (FTIR, Thermo spectrometer), X-ray diffraction (XRD, Philips PW1730, Cu Ka), Brunauer–Emmett–Teller analyses (BET, BELsorp-mini II instrument), and thermogravimetric analysis (TGA, TA Instrument; model SDT Q600, in nitrogen, 50–600 ºC, 10 ºC/min) have been exploited to characterize the nano-carrier.

### Assessment of PersiProtease1 enzymatic activity

PersiProtease1 was screened in silico from tannery wastewater microbiota metagenomic data and cloned, expressed, and purified at the Agricultural Biotechnology Research Institute of Iran (ABRII)^34^. Sequence data for PersiProtease1 have been deposited in GenBank (MW518018). The single PersiProtease1 band was visible, with a molecular weight of 28.5 kDa. Furthermore, the biochemical and structural properties of the enzyme, such as its activity under various harsh conditions (e.g., high temperature, alkaline pH, and high salinity), were thoroughly discussed in our earlier work^34^. The enzyme showed high stability and activity at the interphase of various substrates, suggesting its ability to function efficiently on nanocarriers.

The enzymatic activity of PersiProtease1 was assessed as described previously^36^. A 0.6% casein solution was combined in equimolar amount with PersiProtease1 in a carbonate-bicarbonate buffer (pH 10.0). The mixture was incubated at 60 ℃ for 10 min. The enzymatic reaction was stopped by adding 110 mM trichloroacetic acid (TCA). The mixture was then centrifuged at 10,000 rpm for 10 min to facilitate phase separation.

For the colorimetric analysis, 2.5 mL of a 500 mM Na_2_CO_3_ solution and 0.5 mL of Folin’s phenol reagent were added to 0.5 mL of the resultant filtrate. The mixture was then incubated for 30 min at 37 ℃. By recording the absorbance at 660 nm, the activity of the PersiProtease1 was measured and tyrosine was used as a standard curve. In this context, a unit of protease activity was defined as the enzymatic quantity that liberates 1µmole of tyrosine every minute under the described assay conditions. Bradford assay was used to measure protein content.

### Determination of immobilization efficiency

To measure the efficiency of PersiProtease1 immobilization on CNCs and DCNCs various quantities of CNCs and DCNCs were dispersed in a uniform PersiProtease1 solution, with a concentration set at 0.5 mg/mL (equivalent to 7 U/mg) in 50 mM phosphate buffer at pH 6.0 (at optimal pH of enzyme). The mixture was then incubated for 60 min at a controlled temperature of 25 °C. Subsequently, the samples were centrifuged and washed with phosphate buffer. The samples were then dried under ambient conditions and maintained at 4 °C for subsequent analysis. The evaluation of immobilization efficiency was primarily based on comparing enzyme concentrations before and after the immobilization process following the approach described in previous studies^37^. The supernatant was separated from the immobilization matrix by centrifugation, which facilitated the subsequent measurement of the protein concentration using the Bradford assay^38^. Immobilization efficiency was determined according to the following Eq. (1):

1 $$\text{Immobilization efficiency}\left({\%}\right)=\frac{C_i-C_s}{C_i}\times100$$

In this context, (C_i_) is the initial enzyme concentration, while (C_s_) stands for the enzyme concentration after immobilization.

The relative enzymatic activities of both the free-standing and immobilized variants were measured^34^. This was achieved by measuring the activity of the immobilized enzyme (A) against that of its free form (A_0_):

2 $$\text{Relative activity}\left({\%}\right)=\left(\frac A{A0}\right)\times100$$

This method ensures an in-depth understanding of the efficiency and relative activity of PersiProtease1 when immobilized on nanocarriers.

### Influence of pH and temperature on the activity of the free and immobilized protease

The optimum pH for the free and immobilized enzyme was assayed by investigating enzymatic activities in different pH ranging from 4.0 to 11.0, at 60 ℃ using 50 mM of different buffers including sodium citrate buffer (pH 4.0 and 5.0), potassium phosphate buffer (pH 6.0 to 8.0), and Tris-HCL buffer (pH 9.0 to 11.0). The highest activity was taken as 100% and the relative enzyme activity was calculated^34^.

The effect of temperature on the activity of the free and immobilized enzyme was investigated at 40 to 90 ℃ in 50 mM phosphate buffer at pH 8.0 for PersiProtease1/DCNCs nanoparticle and pH 6.0 for PersiProtease1/CNCs (the optimum conditions of enzymes).

### Kinetic study of the free and immobilized PersiProtease1

Systematic studies were conducted for a comprehensive understanding of the kinetic behavior of both free and immobilized PersiProtease1. The enzymes were exposed to varying concentrations of casein (0.033–1.1 mM) set at 0.6% w/v. Incubation temperature was maintained at 80 ºC. Notably, distinct pH conditions were adopted based on the enzyme variant: a pH of 8.0, which was chosen for PersiProtease1/DCNCs, while a slightly acidic pH (6.0) was employed for PersiProtease1/CNCs.

Subsequently, utilizing the Lineweaver–Burk representation, the paramount kinetic parameters, encompassing the Michaelis − Menten constants (K_m_) and the maximum reaction rate (V_max_), were precisely elucidated.

### Reusability of the immobilized protease

The reusability of both immobilized proteases was determined using 15 consecutive cycles by incubating the enzymes at pH 7.0, at 25 ℃. The immobilized enzymes were then removed from the reaction mixture through centrifugation, washed with 50 mM phosphate buffer pH 7.0, and subsequently used to start a further cycle with a fresh substrate solution. Although the optimal pH for enzymatic activity differs between the free enzyme and its immobilized forms, pH 7.0 was selected to ensure a standardized and neutral environment for comparison. This decision was made to reflect conditions commonly used in industrial applications, where neutral pH is preferred for operational stability across various enzyme systems. The calculated specific activity in the first cycle was considered as 100%.

### Immobilized enzyme leaching studies

For evaluating the leaching of the immobilized PersiProtease1 on CNCs and DCNCs supports, they were incubated at for 30 to 120 min, at 25 °C, and for 1 h at different temperatures ranging from 30 to 80 ℃. The percentage of leaching was investigated by measuring the concentration of protein in the supernatant after centrifugation and its dividing into the extent^9^.

### Statistical analysis

All experiments, including enzyme activity assays, immobilization efficiency tests, stability evaluations, and reusability assessments, were conducted in triplicate to ensure the reliability and reproducibility of the results. The variability between repetitions was carefully monitored and treated statistically. For each set of experiments, the standard deviation (SD) was calculated and reported alongside the mean values to represent the degree of variability observed in the data. These calculations were conducted using the software, Microsoft Excel.

## Results and discussion

### Characterization of nanocarriers

**Figure 1:**
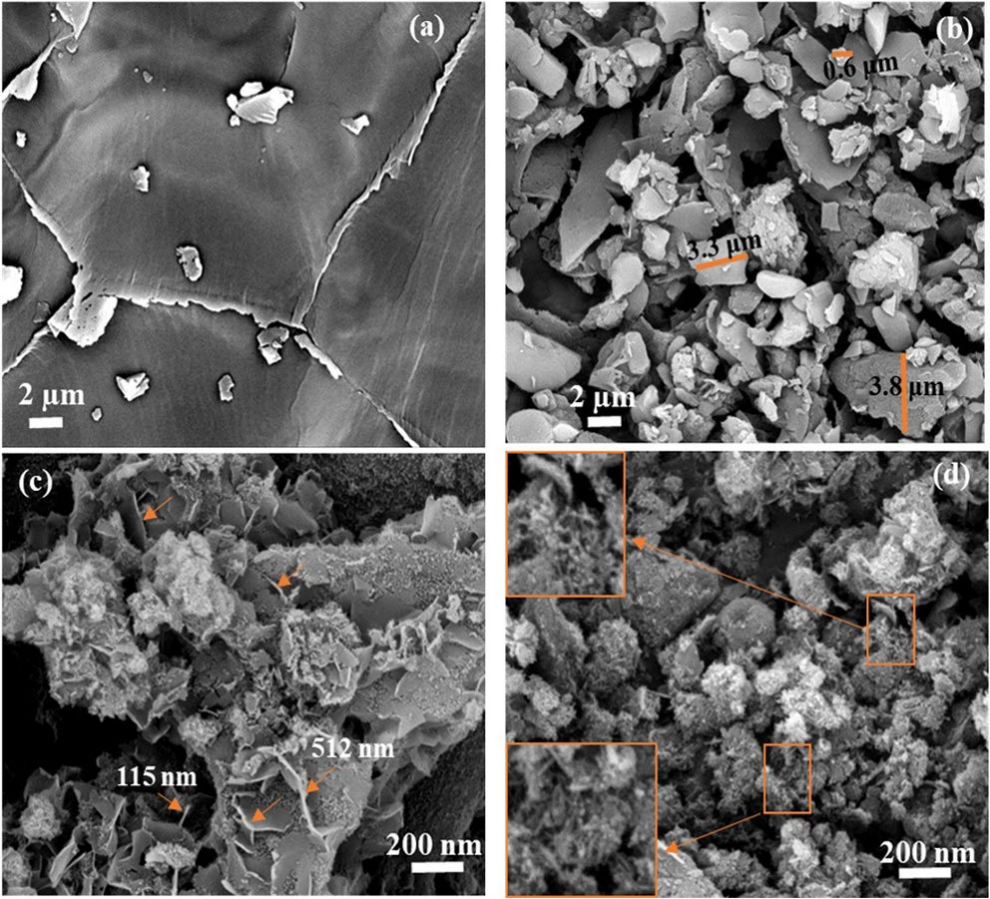
SEM images of (**a**) raw QS, (**b**) NaOH-treated QS, (**c**) CNCs, and (**d**) DCNCs samples.

SEM images of raw QS, NaOH-treated QS, CNCs, and DCNCs are compared and contrasted in Fig. 1. While the raw QS showed an almost smooth surface containing a number of micrometric hunks, the NaOH-treated QS sample displayed many stacked and chopped lumps with micrometric sizes ranging from (0.5–4 μm) (Fig. 1a, b). This confirmed that the husks were crushed and disintegrated during the alkali treatment, which could possibly expedite cellulose fibrillation through disruption of the lignin or hemicellulose binders and facilitate cellulose nanofiber production from QS in the next chemical steps^39^. SEM images of the CNC sample revealed that the intact surfaces of chopped lumps in NaOH-treated QS were disrupted under oxidative conditions, and many slender rod-like particles (diameter below 20 nm and length between 100 and 500 nm) were observed in the image. In fact, during the oxidation reaction, KMnO_4_ (strong oxidant) in the diluted sulfuric acid medium was reduced and Mn^3+^ and MnO_4_^−^ were generated, both of which are reactive species and able to oxidize the amorphous parts of cellulose and other binder materials. Oxalic acid in this system could form a complex with Mn^3+^ [Mn(C_2_O_4_^2−^)]^+^ and stabilized this ion to prevent its back reduction to Mn ion^40^. Because of the strong oxidizing capacity of this system almost all the remaining non-cellulosic constituents and amorphous cellulosic regions were removed along with the oxidation of crystalline parts of cellulose which resulted in breaking of the large lumps into acicular carboxylated-NC particles (Fig. 1c). After functionalization with DCC, numerous small needle-like CNCs were prepared which mostly aggregated in the SEM image of DCNC sample (Fig. 1d). To scrutinize the morphology of the latter two samples more powerful microscopy instrument is needed.

**Figure 2:**
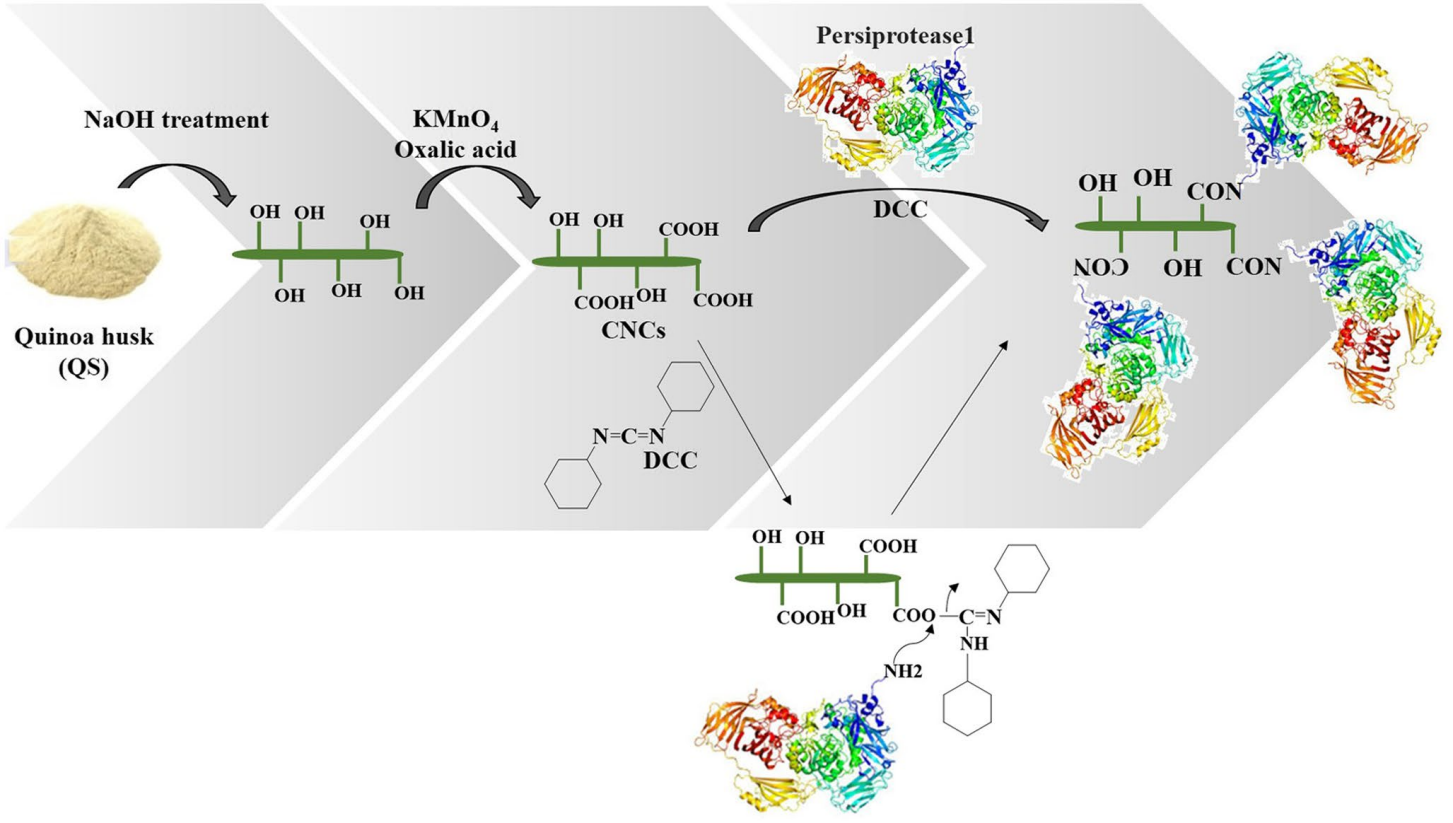
Schematic illustration of preparation of CNCs from quinoa husk and mechanism involved in covalent immobilization of Persiprotease1 onto DCC-activated CNCs (DCNCs).

The reaction mechanisms involved in the immobilization of the enzyme on nanocarrier through covalent bonding using DCC have been well studied in the previous reports^41–45^. Firstly, carboxylic acid functionalities on CNCs were transformed into different reactive esters using DCC as the activating groups. During the last decades, carbodiimides have been extensively reported as activating groups to modify the acids^41–45^. Activated carboxyl moiety then react with amine groups in the enzyme to provide covalent bonding between the carrier and enzyme (Fig. 2). Carbodiimides including N, N′-Dicyclohexylcarbodiimide (DCC), N, N′-Diisopropylcarbodiimide (DIC) and 1- ethyl-3-(3-dimethyl-aminopropyl)carbodiimide (EDC) are commonly used in the covalent conjugation of proteins to the supports containing carboxylic acid functionalities^41^.

**Figure 3:**
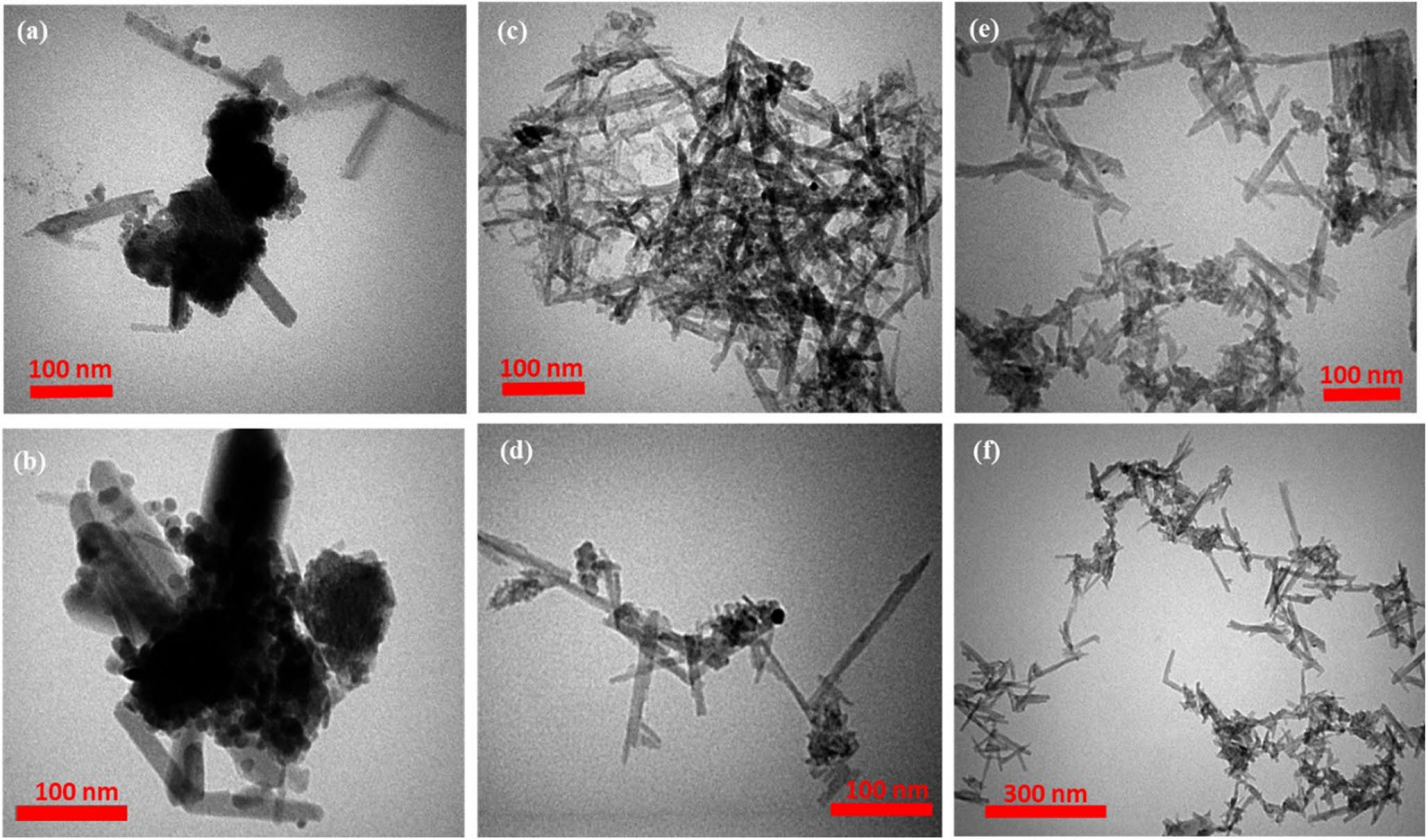
TEM images of (**a**, **b**) NaOH-treated QS, (**c**, **d**) CNCs, and (**e**, **f**) DCNCs samples.

TEM images of NaOH-treated QS, CNCs, and DCNCs are shown in Fig. 3. After NaOH treatment, although some rod-like particles (length of 150–170 nm, diameter of approximately 20 nm) could be seen in the images, the presence of larger lumps indicated that alkali treatment was inadequate for the complete degradation of raw QS and production of cellulose nano-fibers (Fig. 3a, b). The fabrication of rod-like NCs was confirmed by TEM analysis of CNCs (Fig. 3c, d), and the sharp edges at the rod tips indicated the absence of hanging carboxylated chains floating in their surrounding medium^46^. TEM images of the DCNCs sample confirmed that the rod structures of the NCs were well maintained after functionalization with DCC (Fig. 3e, f). The average diameter of the nanorods was 10 nm in both samples, however, the CNC sample had longer nanorods than the DCNC sample (average length of 103 nm vs. 76 nm for CNCs and DCNCs, respectively). TEM images confirmed the morphological changes from the bulk materials in NaOH-treated QS toward cellulosic nano-rods of CNCs and DCNCs. Although some clumping/aggregations could be seen CNCs and DCNCs (possibly due to the sample preparation procedure for imaging), this did not affect the properties of the nanocarriers. To confirm that the specific surface areas of these samples were analyzed, the results showed a clear gap between the bulk materials (raw QS, NaOH-treated QS) and nanocellulose materials (CNCs and DCNCs). Moreover, it was suggested that nanocarriers solution samples could be prepared using an ultrasonic homogenizer (sonicator) instead of an ultrasonic bath to avoid clumping/aggregation.

**Figure 4:**
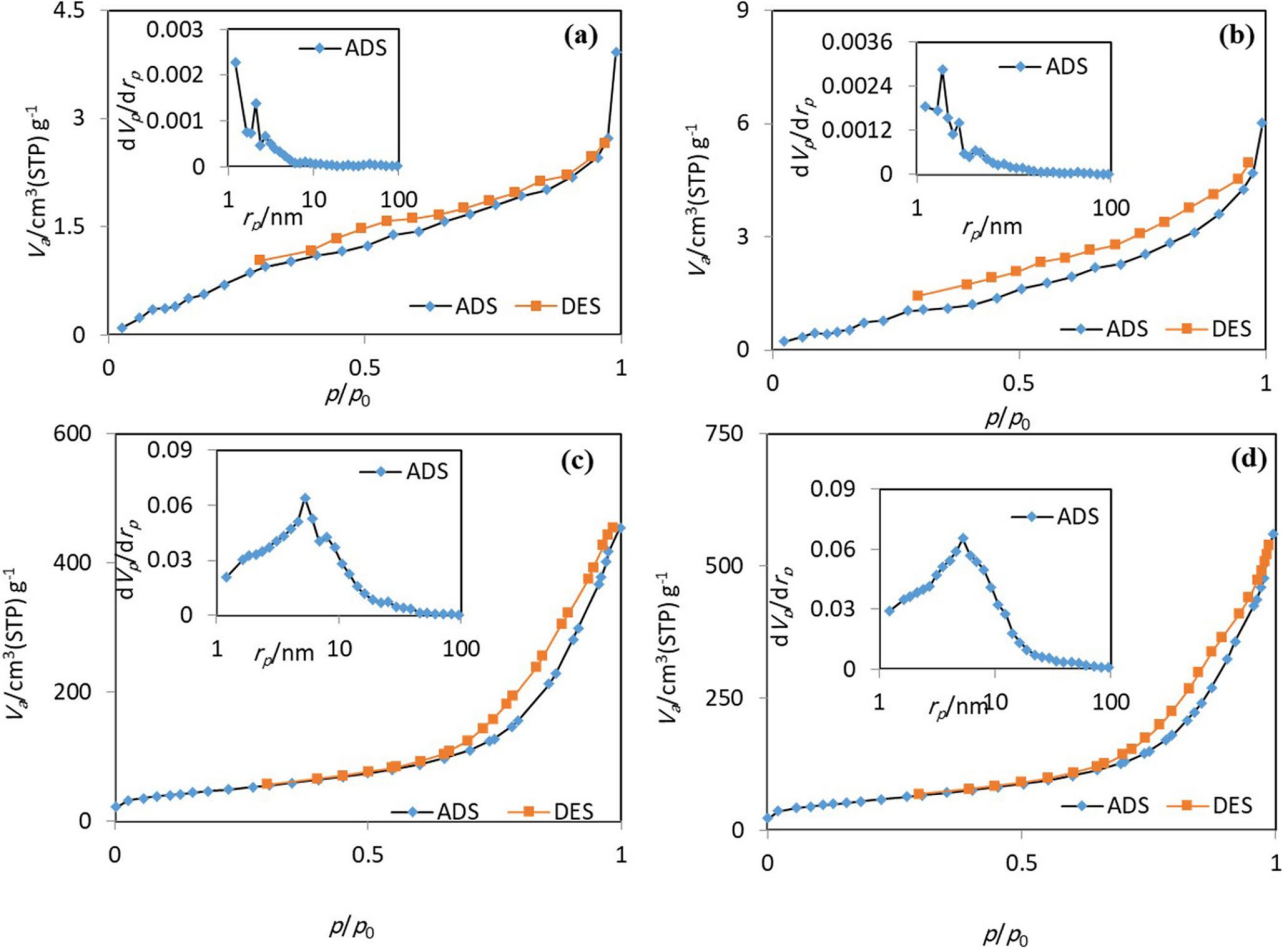
Nitrogen adsorption/desorption isotherms and the pore size distributions (inset) of (**a**) raw QS, (**b**) NaOH-treated QS, (**c**) CNCs, and (**d**) DCNCs samples.

In order to investigate the porosity and specific surface area of the as-synthesized samples, BET analyses were conducted (Fig. 4; Table 1.). The samples displayed hysteresis loops of type-IV and III isotherms meaning monolayer-multilayer N_2_ adsorption on mesoporous structure (pore diameter size of 2 to 50 nm) with slit-shaped pores^47^. The measured BET surface area of 5.38, 6.22, 173.58 and 204.46 m^2^/g were found for raw QS, NaOH-treated QS, CNCs, and DCNCs, respectively (Table 1.). Although alkali treatment did not remarkably change the surface area and pore structure of raw sample, oxidation reaction resulted in a significant increment (more than 25-fold, from 6.2 to 173 m^2^/g) of surface area of the sample. Besides, DCC-functionalization lead to an increase of the surface area up to 204 m^2^/g in DCNCs sample. Such mesoporous structure with high surface area would possibly provide easy access of enzymes and facilitated the immobilization process on these carriers.

**Table 1:** Comparison of BET analyses of raw quinoa husk (QS), alkali treated quinoa husk (NaOH treated-QS), carboxylated nanocellulose **(**CNCs), and CNCs functionalized with DCC (DCNCs).

***Sample***	***Raw QS***	***NaOH treated-QS***	*CNCs*	***DCNCs***
***BET surface area (m*** ^***2***^ ***/g)***	*5.3801*	*6.2165*	*173.58*	*204.46*
***Pore volume (cm*** ^***3***^ ***/g)***	*0.00598*	*0.00901*	*0.6823*	*0.8338*
***Mean pore diameter (nm)***	*4.4452*	*8.547*	*15.722*	*16.312*

The investigation leverages a green, efficient, and feasible approach to oxidize low-cost agricultural waste, quinoa husk, for the synthesis of CNC. This presents a novel method of valorizing agricultural by-products which are otherwise considered waste, thus contributing to sustainability and waste reduction.

### Persiprotease1 immobilization on CNCs and DCNCs nanocarriers

**Figure 5:**
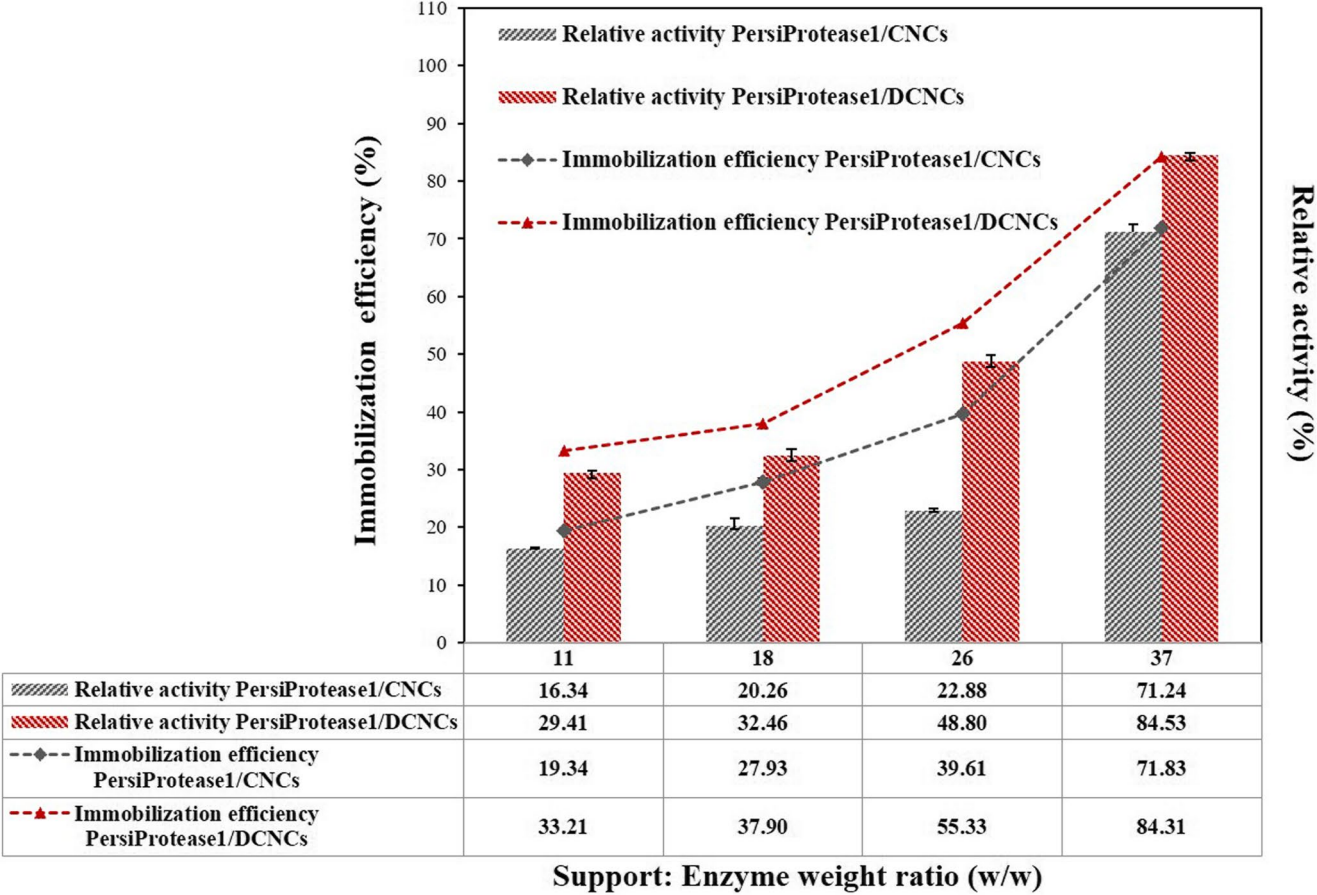
Immobilization efficiency (%) and relative activity (%) of the PersiProtease1/CNCs and PersiProtease1/DCNCs.

To determine the best Support: Enzyme weight ratios (w/w), the immobilization efficiency of PersiProtease1 was investigated by soaking various amounts of CNCs and DCNCs in the enzyme solution. As shown in Fig. 5, the maximum immobilization efficiency and relative activity for both carriers were enhanced as the Support: Enzyme weight ratio increased and reached the maximum value at a ratio of 37. This phenomenon can be attributed to the more accessible active sites provided at higher dosages of nanocarriers and the development of interactions with protease^9,10^. From the other point of view, the covalently bound enzyme through nucleophilic substitution (PersiProtease1/DCNCs) displayed higher immobilization efficiencies and relative activities rather than physically immobilized enzyme (PersiProtease1/CNCs). This could be attributed to the fact that enzyme adsorption through electrostatic attractions between the enzyme and its nanocarrier results in weaker conjugation and lower efficiencies than the covalent immobilization of the enzyme^48^.

### Determination of kinetic constants for free and immobilized proteases

The kinetic study of the free and immobilized PersiProtease1 was carried out using casein as substrate. According to the results, the K_m_ of free enzyme was found to be 0.05$\:\pm\:$0.06 mM and this value reduced in immobilized protease to 0.04$\:\pm\:$0.03 and 0.03$\:\pm\:$0.07 mM for PersiProtease1/DCNCs and PersiProtease1/CNCs, respectively. This indicated the enhancement of enzyme affinity toward substrate through immobilization and confirmed improved catalytic activity of enzyme^49^. Besides, the V_max_ of the immobilized PersiProtease1 was approximately tripled compared to the free enzyme, showing V_max_ of 0.42 $\:\pm\:$0.11, 0.41$\:\pm\:$0.20, and 0.15$\:\pm\:$0.13 U/mg for PersiProtease1/DCNCs, PersiProtease1/CNCs, and free PersiProtease1, respectively. These outcomes highlighted the efficiency and effectiveness of applied support for protease immobilization. Similarly, protease from *Bacillus* demonstrated higher a V_max_ as well as a higher affinity to the substrate after immobilization onto hollow core-mesoporous shell silica^50^. In another study, immobilization of protease from *Bacillus brevis* on Ca-alginate led to higher V_max_ and lower K_m_ of enzyme^51^. Moreover, because the V_max_ value referred to the maximum reaction rate, when all the active sites of the enzyme are saturated with substrate^48^, So, the almost similar V_max_ values for both immobilized enzymes confirmed that the differences in the strategies of enzyme immobilization did not change the diffusional constrains and steric hindrance of the active site of enzyme by the nanocarrier^48,52^.

The comparative analysis between covalent and physical immobilization of the PersiProtease1 enzyme on the CNCs provides valuable insights into the superior performance of covalently immobilized enzymes. This pioneering approach, underscored by a detailed kinetic and stability analysis, lays a solid foundation for the potential application of synthesized CNCs in biocatalysis and other biotechnological processes. This study significantly contributes to the broader endeavor of promoting sustainability in scientific practices while pushing the frontiers in enzyme immobilization technologies.

### Performance of free vs. immobilized protease across various temperatures and pH values

**Figure 6:**
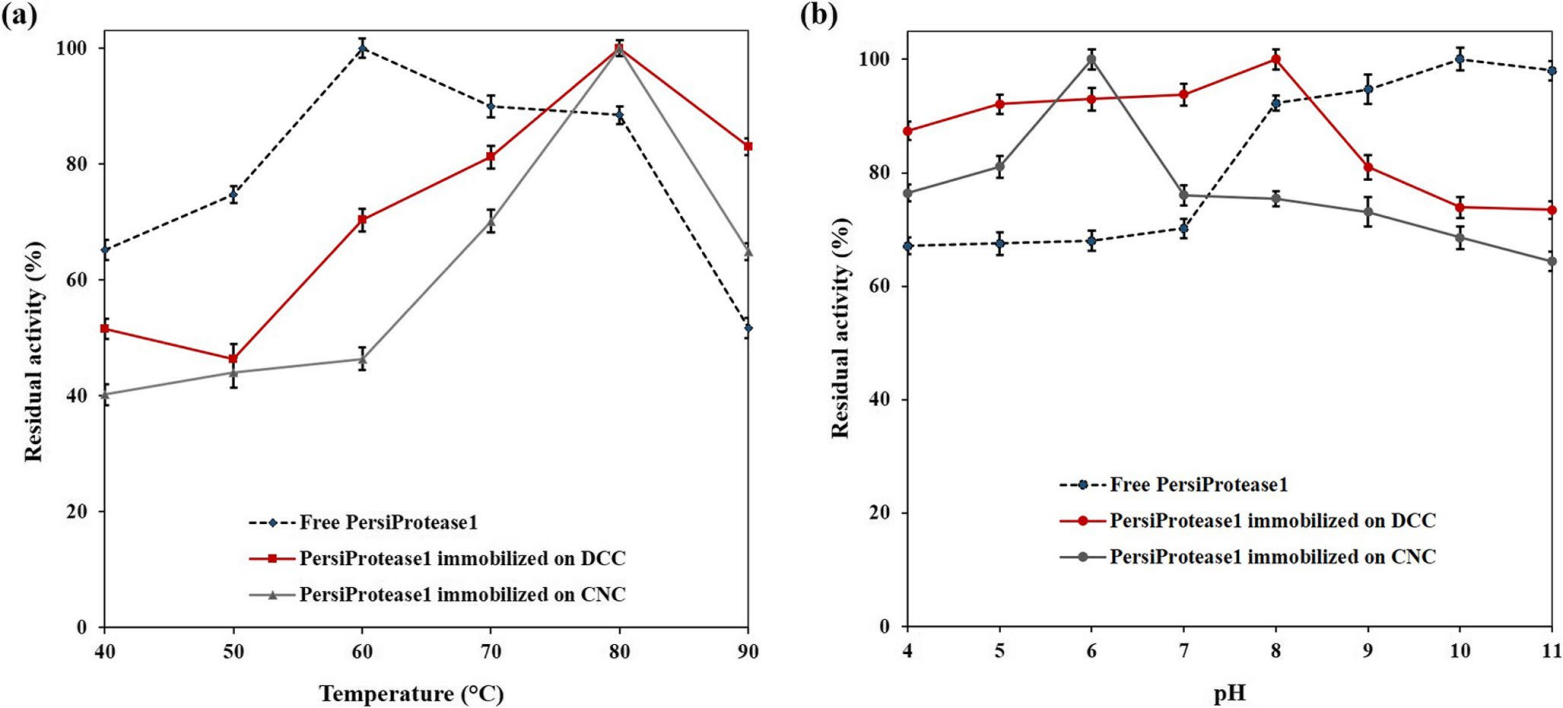
Residual activity of the free and immobilized PersiProtease1 incubated at (**a**) different temperatures from 40 ℃ to 90 ℃ and (**b**) pH values ranging from 4.0 to 11.0.

The activities of free and immobilized enzymes were evaluated over a broad range of temperatures and pH values (Fig. 6). The free enzyme exhibited the highest residual activity at 60 ºC, and this value declined up to 88%, at 80 ºC, and reached 51%, at the highest temperature of 90 ºC. In contrast, the immobilization of PersiProtease1 on both nanocarriers shifted the temperature to 80 ºC. Meanwhile, the immobilization technique can increase protease activity at elevated temperatures. In other words, the free enzyme lost half of its activity at 90 ºC, whereas PersiProtease1/DCNCs revealed 83% activity under this temperature and this value reached 65% for PersiProtease1/CNCs. This indicated that the immobilization of PersiProtease1 led to higher enzyme stability at extreme temperatures (Fig. 6a). The higher thermal stability of the covalently immobilized enzyme compared to physically immobilized and free enzymes at elevated temperatures could be attributed to greater rigidity of enzyme’s conformation and higher activation energy required for unfolding the covalently immobilized enzyme^53^. Although the immobilized enzymes exhibited advantages at extreme temperatures, they are less active than free enzyme at mild temperatures. In brief, the enhanced stability of immobilized enzymes at elevated temperatures could be attributed to the restricted conformational flexibility resulting from immobilization, which helps the enzyme maintain its active form under stress conditions. However, at mild temperatures, this rigidity hinders the flexibility needed for optimal catalytic performance, leading to lower activity compared to the free enzyme^54^. The free enzyme, owing to its natural flexibility, interacted more efficiently with substrates at lower temperatures. Furthermore, the covalent bonding of PersiProtease1 to the DCNC nanocarrier may introduce steric hindrance and diffusional constraints that slightly reduce catalytic efficiency at lower temperatures compared to free enzymes.

Moreover, the influence of pH of the medium on the activity of free and immobilized PersiProtease1 was examined (Fig. 6b). The catalytic activity of the free enzyme was enhanced as the pH increased, and showed maximum activity at pH 10.0. The free enzyme demonstrated lower than 70% activity at pH ranges from 4.0 to 7.0. In contrast, immobilization of PersiProtease1 on both nanocarriers significantly increased the residual activity of the enzyme under acidic conditions. Using DCNC as a support for enzyme manifested 87–93% enzymatic activity at pH 4.0 to 7.0, and showed the highest activity (100%) at pH 8.0. Meanwhile, a shift in pH optima from 10.0 to 8.0 was observed by immobilization of the enzyme on this nanocarrier. Furthermore, when CNCs were used for the immobilization of PersiProtease1, a remarkable shift in the optimum pH toward the acidic region was observed. PersiProtease1/CNCs exhibited more than 76% enzymatic activity from pH 4.0 to 7.0, and reached maximum activity (100%), at pH 6.0. These results showed that enzyme activity was higher under acidic or neutral conditions after immobilization on DCNCs and CNCs nanocarriers. These results are in accordance with previous studies that immobilized protease from *Salipaludibacillus agaradhaerens* on mesoporous core-shell nanospheres and thermostable protease from a haloalkaliphilic bacteria^17,24^.

### Reusability of immobilized PersiProtease1 enzymes

**Figure 7:**
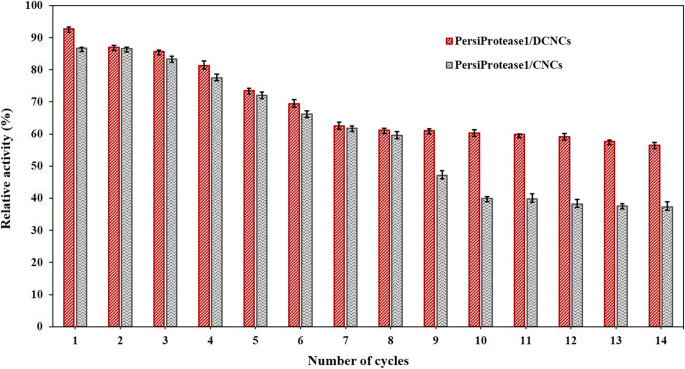
Reusability of PersiProtease1 immobilized on DCNCs and CNCs nanocarriers.

The reusability of immobilized enzymes is a crucial factor in the industrial application of enzymes and the development of novel biotechnology^55^. In the current study, the reusability of immobilized PersiProtease1 was studied over 15 cycles, and the results are illustrated in Fig. 7. The PersiProtease1/DCNCs retained 92.72% of their initial activity in the second run, and this value decreased gradually to 61% in the 9th cycle, followed by almost constant values in the further runs and reached 56.52% at the final 15th cycle, which revealed good operational stability of the enzyme through immobilization. Similarly, PersiProtease1/CNCs exhibited nearly 60% relative activity after the 9th cycle, but this value declined in the following cycles and reached 37.28% in the final run. The downward trend observed in the reusability study of the immobilized enzyme on the nanocarriers could be due to enzyme leaching during incubation, washing of the substrate and distortion of enzyme conformation within the reutilization^48^. These results confirmed the strong activity of the immobilized PersiProtease1 after 15 cycles which demonstrated the high performance of the immobilized enzymes. Similarly, immobilization of a halophilic Bacillus protease on silica nanoparticles and a protease from *P. vannamei* showed high activity after 6 and 10 cycles^4,56^. Moreover, the higher reusability of covalently bound PersiProtease1 (PersiProtease1/DCNCs) compared to its physically immobilized form confirmed that covalent attachment could provide better prevention of enzyme deformation or leaching^48^.

As mentioned above, the reusability study results could provide data about the effectiveness of nanocarriers for enzyme immobilization in long-term applications, which is essential for understanding the durability and viability of the application. Retaining more than 56% and 37% enzyme activity after 15 cycles, for DCNCs and CNCs immobilized PersiProtease1, respectively, could highlight the durability and viability of the immobilized enzyme, confirming their potential for long-term applications in industrial settings. This study further delves into the comparison of covalent and physical immobilization of the PersiProtease1 enzyme on CNCs, revealing significantly enhanced enzyme stability, especially at elevated temperatures, and reusability when covalently immobilized. This demonstrates the potential of this novel nanocarrier to improve enzyme performance, which is of great significance in biotechnological applications.

### Leaching studies

The immobilized PersiProtease1 on DCNC and CNC nanocarriers was subjected to leaching analysis to confirm the potency of enzyme conjugation (Table 2). Based on these results, the leaching percentage increased as the temperature and time increased. This result indicated that higher temperatures or longer times led to impaired enzyme-support interactions and that a higher amount of enzyme might be leached out from the support. This can be explained by the fact that thermal energy can break the weak non-covalent attachments between the enzyme and the support^57,58^. Compared to CNC, the DCNC support demonstrated a lower enzyme leaching percentage, indicating stronger electrostatic attractions between the enzyme and carrier, and showed higher stability without much enzyme loss. Furthermore, the leaching experiment results could provide data on the stability of immobilized enzymes on nanocarriers in long-term applications. The results demonstrated that enzyme stability was strongly correlated with temperature and incubation time, and the DCNC nanocarrier showed superior performance, exhibiting lower enzyme leaching (as low as 2.63% at 30 °C and 11.68% at 80 °C than the CNC nanocarrier. This indicates that covalent immobilization via DCNCs provided greater enzyme retention and higher stability at elevated temperatures​ and longer times.

**Table 2:** Enzyme leached out (%) at various incubation temperatures and time from immobilized PersiProtease1 on DCNC and CNC nanocarriers.

		Temperature (°C), 1 h						Time (min), 25 °C			
Enzyme leached (%)	**Support**	30	40	50	60	70	80	30	60	90	120
	**CNC**	3.15	5.81	8.02	11.03	13.80	17.14	1.06	3.64	4.79	7.57
	***DCNC***	*2.63*	*4.09*	*5.60*	*7.56*	*10.41*	*11.68*	*0.32*	*1.36*	*3.51*	*6.90*

This study demonstrates a successful example of enhancing the stability and performance of PersiProtease1 immobilization. This is significant, as it may pave the way for broader applications of CNCs in enzyme immobilization and other biocatalytic processes.

### Industrial development: advantages and challenges

In the current study, we attempted to combine nanobiotechnology and green chemistry to develop a renewable cellulose-based nanocarrier to enhance the efficiency of protease enzymes. More specifically, we followed a low-cost method for the synthesis of carboxylated nanocellulose from an agricultural waste under mild reaction conditions (50 °C, 1 w% H_2_SO_4_ medium) using KMnO_4_ and H_2_CO_4_ reagents, with high yields. The oxidant dosage in this approach is much lower and cheaper than that in the conventional Tempo method and approximately half of the weight% of quinoa husk was converted to CNC. Compared to the most commonly used methods to prepare CNCs (sulfate acid hydrolysis and TEMPO-mediated oxidation), the current strategy is more cost-effective and environmentally friendly^40^. For example, the sulfuric acid hydrolysis method requires too much sulfuric acid (> 60% concentration) and it is easily cause acid pollution in the environment. Moreover, the cost of recycling sulfuric acid is very high, and the surface of carboxylated nanocellulose containes COOH functionalities, which are easier to modify, such as taking part in covalent crosslinking and grafting reactions. Moreover, TEMPO-mediated oxidation requires pH values of 10 ~ 11 during the entire oxidation process, toxic TEMPO reagents would result in environmental concerns, and the TEMPO-oxidized cellulose product requires post-treatment with NaBH_4_ to reduce the aldehyde and ketone groups present in the oxidized cellulose to hydroxyl groups^40^. According to this background, the synthesis of CNCs on a large scale for enzyme immobilization applications might be advantageous, but more study is needed to estimate the detailed production costs accurately at the industrial level.

One may ask about the environmental impacts of the entire nanocarrier production and disposal process. In this regard, mild chemical processes, along with the biodegradable nature of the nanocellulose carriers, contribute to minimizing the environmental footprint. While the use of quinoa husks as a source of nanocellulose offers clear environmental advantages by repurposing agricultural waste, it is essential to consider the environmental impacts of the entire production and disposal process. The production of CNCs and DCNCs from quinoa husks involves relatively mild chemical treatments with sodium hydroxide, potassium permanganate, and oxalic acid. These chemicals, which are widely available and manageable in industrial settings, require handling to mitigate their environmental impacts. For example, KMnO_4_ is a green and industrial oxidant reagent with a relatively low cost, easy handling, effectiveness, and comparative stability over a wide pH range. The effluents generated during the chemical oxidation process can be neutralized and treated using standard wastewater treatment practices, which minimizes the environmental footprint of the production process^59^. Moreover, the nanocellulose-based carriers themselves are biodegradable, making them environmentally friendly in terms of disposal. After the enzyme has been used and the nanocarriers are no longer viable, CNCs and DCNCs can break down naturally in the environment without contributing to long-term pollution, unlike synthetic polymers. This biodegradability reduces the potential environmental impact of disposal^60^. Further research could focus on optimizing the process to reduce chemical use and energy consumption and enhance the overall sustainability of the nanocarrier production cycle.

For further clarification of the advantages and disadvantages of the proposed approach compared to other technologies available in the market for enzyme immobilization, it should be mentioned that among of the many studies that report protease immobilization on various nanocarriers, there are a few studies that claim their application on a commercial scale. This is due to some imperative factors, including: (1) stability of the enzyme should remain high during the intermediate steps, (2) production protocols should be cost-effective, scalable, and reproducible, and (3) processes should have minimal environmental effects^33^. However, to provide a comprehensive view of the advantages and potential limitations of the current study compared with other available immobilization technologies, the most commonly used immobilization techniques are compared and contrasted here. (i) Physical adsorptionis one of the simplest and most cost-effective immobilization methods. However, the primary drawback of physical adsorption is the weak binding between the enzyme and support, which often results in enzyme leaching during repeated use, especially in industrial processes. In contrast, the covalent bonding utilized in this study with DCNC nanocarriers provides stronger and more stable attachment, reduces enzyme leaching and improves the reusability of the immobilized enzyme. (ii) Ionic binding, which offers moderate binding strength. However, it can be highly sensitive to environmental conditions such as pH changes and ionic strength, which may cause enzyme desorption. The covalent approach used in this study minimized such desorption risks, ensuring that the enzyme remained attached even under variable conditions. (iii) Entrapment and Encapsulation can protect enzymes from environmental factors. Nevertheless, they often suffer from diffusion limitations, where substrates or products cannot easily pass through the encapsulating material. The nanocellulose-based supports used in this study offer a large surface area and minimal diffusional resistance, allowing for efficient substrate-enzyme interactions. (iv) Synthetic Polymer Supports (i.e., polyacrylamide, polyethylene glycol, and polystyrene) are widely used for enzyme immobilization. Although they provide mechanical strength and stability, they are often non-biodegradable and expensive. CNCs and DCNCs derived from agricultural waste are biodegradable and environmentally friendly, making them preferable options for sustainable industrial applications^61^. By comparing the advantages and disadvantages of other immobilization technologies, it is evident that the approach used in this study, particularly covalent immobilization on DCNCs, may provide a balance of stability, reusability, and environmental sustainability, which makes it competitive with other available technologies.

Discussing how the process of synthesizing nanocarriers and immobilizing enzymes can be scaled up for industrial production requires explanations from three different viewpoints. (1) Nanocarrier synthesis scalability: The synthesis of CNC from quinoa husks follows a straightforward and scalable method that uses readily available reagents, such as potassium permanganate, and oxalic acid. These chemicals are inexpensive and can be easily obtained in large quantities, which makes this process economically feasible for industrial production. Additionally, the reaction conditions (e.g., mild temperatures and pressures) ensure that the process can be easily scaled up with standard industrial equipment, such as large-scale reactors and centrifuges, without requiring specialized or expensive infrastructure. (2) Enzyme immobilization scalability: The immobilization of PersiProtease1 onto CNC and DCNC nanocarriers was performed via physical and covalent attachment, both of which are amenable to scaling. Physical immobilization involves simple mixing, whereas covalent attachment relies on the use of NN′-dicyclohexylcarbodiimide, which is a common coupling agent used in industrial-scale biocatalyst production. Both immobilization strategies require minimal energy input and can be executed in batch or continuous-flow systems, which are standards in industrial biotechnology. (3) Process Optimization for Industrial Implementation: To optimize these processes for industrial settings, we propose the use of high-shear mixers and large-scale filtration systems to enhance the uniformity of nanocarrier production and enzyme immobilization. Additionally, automation of the mixing and washing steps can increase throughput and reduce labor costs. Given the stability and reusability of the immobilized enzymes demonstrated in our study (e.g., retaining 56% activity after 15 cycles), these systems are well suited for prolonged industrial applications, minimizing downtime and maximizing efficiency.

Although the focus of this study was primarily on the immobilization and activity of PersiProtease1 under controlled laboratory conditions, it is important to acknowledge that real-world industrial or environmental samples may contain various interfering substances that could affect the activity of immobilized enzymes. In practical applications, components such as salts, organic solvents, heavy metals, and other proteinaceous substances could potentially interfere with enzyme activity by altering the conformation of the enzyme, competing for active sites, or causing partial deactivation of the enzyme. Previous studies on enzyme immobilization have shown that immobilized enzymes tend to have higher tolerance to such interference than free enzymes, owing to the stabilization provided by the immobilization matrix^62^. However, to further validate the applicability of immobilized PersiProtease1 in real-solutions, it would be beneficial to conduct additional tests with complex matrices, such as industrial effluents or real samples containing salts, detergents, and organic solvents. Moreover, possible adverse or unwanted reactions in certain environments or with certain substrates, which may result from enzyme immobilization, should be further considered.

Finally, this should be mentioned that since the research has been carried out on a lab scale to examine the suitability of the CNC-based nanocarriers and the method of the immobilization for protease enzymes, the results could not be generalized for industrial applications. Additional research studies are required for the integration of the present study into industrial processes, examining the proficiency of the immobilized enzyme in the real samples, and evaluating possible adverse reactions with specific substrates, which all are the ongoing and/or future research plans of this research team.

## Conclusion

The strategic immobilization of protease enzymes amplifies their reusability, offering pronounced economic advantages in industrial applications, and heralding new horizons for bespoke utility. The novelty of this work primarily resides in the utilization of an efficient, and feasible synthesis approach to transform a low-cost agricultural by-product into CNC, a robust nanocarrier for protease immobilization. This innovative synthesis method is significant as it aligns with the global shift towards eco-friendly and sustainable scientific practices. This not only presents a sustainable method for valorizing agricultural wastes but also introduces a novel nanocarrier that could significantly improve enzyme performance, particularly in terms of thermal stability and reusability. These uniquely fashioned rod-like CNCs functionalized with DCC served as nanocarriers for PersiProtease1, covalently bonded through nucleophilic substitution. The immobilized enzymes exhibited increased thermal stability, an acidic pH shift, and Vmax enhancement compared to the free enzyme. Furthermore, the main hypothesis of the research was confirmed since the covalently immobilized enzyme showed higher reusability than physically immobilized enzymes, highlighting the efficiency of this approach. Besides, minimal enzyme leaching across varied incubation conditions further confirmed the efficacy of covalent conjugation methodology. This study serves as a cornerstone in the realm of protease immobilization, accentuating the latent potential of agricultural remnants as eco-conscious feedstock for enzyme immobilization and crafting pathways for future scholarly exploration in the domain.

## Data Availability

All data generated or analyzed during this study are included in this published article.

## References

[1] Cavalcante, F. T. T. et al. Designing of nanomaterials-based enzymatic biosensors: synthesis, Properties, and applications. *Electrochem* **2**, 149–184 (2021).

[2] Cavalcante, A. L. G. et al. Advancements in enzyme immobilization on magnetic nanomaterials: toward sustainable industrial applications. *RSC Adv.* **14**, 17946–17988 (2024). 38841394 10.1039/d4ra02939a PMC11151160

[3] Singh, R. S., Singh, T. & Pandey, A. Microbial enzymes-an overview. In: *Biomass, Biofuels, Biochemicals: Advances in Enzyme Technology*. (2019). 10.1016/B978-0-444-64114-4.00001-7

[4] Shojaei, F., Homaei, A., Taherizadeh, M. R. & Kamrani, E. Characterization of biosynthesized chitosan nanoparticles from Penaeus vannamei for the immobilization of P. Vannamei protease: an eco-friendly nanobiocatalyst. *Int. J. Food Prop.* **20**, 1413–1423 (2017).

[5] Kirupa Sankar, M., Ravikumar, R., Naresh Kumar, M. & Sivakumar, U. Development of co-immobilized tri-enzyme biocatalytic system for one-pot pretreatment of four different perennial lignocellulosic biomass and evaluation of their bioethanol production potential. *Bioresour Technol.* **269**, 227–236 (2018). 30179756 10.1016/j.biortech.2018.08.091

[6] Lu, J., Nie, M., Li, Y., Zhu, H. & Shi, G. Design of composite nanosupports and applications thereof in enzyme immobilization: a review. *Colloids Surf. B Biointerfaces*. **217**, 112602 (2022). 35660743 10.1016/j.colsurfb.2022.112602

[7] Verma, M. L., Naebe, M., Barrow, C. J. & Puri, M. Enzyme immobilisation on amino-functionalised Multi- Walled Carbon nanotubes: structural and biocatalytic characterisation. *PlosOne*. **8**, 16–18 (2013). 10.1371/journal.pone.0073642 PMC3772012 24069216

[8] Singh, N., Dhanya, B. S. & Verma, M. L. Nano-immobilized biocatalysts and their potential biotechnological applications in bioenergy production. *Mater. Sci. Energy Technol.* **3**, 808–824 (2020).

[9] Ariaeenejad, S., Hosseini, E., Motamedi, E., Moosavi-Movahedi, A. A. & Salekdeh, G. H. Application of carboxymethyl cellulose-g-poly(acrylic acid-co-acrylamide) hydrogel sponges for improvement of efficiency, reusability and thermal stability of a recombinant xylanase. *Chem. Eng. J.* **375**, 122022 (2019).

[10] Motamedi, E. et al. Upgrading the enzymatic hydrolysis of lignocellulosic biomass by immobilization of metagenome-derived novel halotolerant cellulase on the carboxymethyl cellulose-based hydrogel. *Cellulose*. **8**, 3485–3503 (2021).

[11] Jackson, E., Correa, S. & Betancor, L. Cellulose-based nanosupports for enzyme immobilization. In: *Cellulose-Based Superabsorbent Hydrogels* 1235–1253. Springer Nature Switzerland, (2019).

[12] Ariaeenejad, S., Motamedi, E. & Hosseini, G. Highly efficient removal of dyes from wastewater using nanocellulose from quinoa husk as a carrier for immobilization of laccase. *Bioresour Technol.* **349**, 126833 (2022). 35149184 10.1016/j.biortech.2022.126833

[13] Ariaeenejad, S., Kavousi, K., Maleki, M. & Motamedi, E. Application of free and immobilized novel bifunctional biocatalyst in biotransformation of recalcitrant lignocellulosic biomass. *Chemosphere*. **285**, 131412 (2021). 34329139 10.1016/j.chemosphere.2021.131412

[14] Ariaeenejad, S., Motamedi, E. & Hosseini, G. Immobilization of enzyme cocktails on dopamine functionalized magnetic cellulose nanocrystals to enhance sugar bioconversion: a biomass reusing loop. *Carbohydr. Polym.* **256**, 117511 (2021). 33483032 10.1016/j.carbpol.2020.117511

[15] Naveed, M. et al. Protease—A versatile and ecofriendly Biocatalyst with Multi-industrial Applications: an updated review. *Catal. Lett.* **151**, 307–323 (2021).

[16] Li, Q., Yi, L., Marek, P. & Iverson, B. L. Commercial proteases: present and future. *FEBS Lett.* **587**, 1155–1163 (2013). 23318711 10.1016/j.febslet.2012.12.019

[17] Ibrahim, A. S. S. et al. Stabilization and improved properties of salipaludibacillus agaradhaerens alkaline protease by immobilization onto double mesoporous core-shell nanospheres. *Int. J. Biol. Macromol.* **166**, 557–566 (2021). 33186653 10.1016/j.ijbiomac.2020.10.213

[18] Liu, X. & Kokare, C. *Microbial Enzymes of Use in Industry. Biotechnology of Microbial Enzymes: Production, Biocatalysis and Industrial Applications* (Elsevier Inc., 2017). 10.1016/B978-0-12-803725-6.00011-X

[19] Alves, L. D. F. et al. Metagenomic approaches for understanding new concepts in microbial science. *Int. J. Genomics* **2018**. 1–15 (2018). 10.1155/2018/2312987 PMC6126073 30211213

[20] Ariaeenejad, S. et al. Highly efficient computationally derived Novel Metagenome α-Amylase with robust stability under extreme denaturing conditions. *Front. Microbiol.* **12**, 1–14 (2021). 10.3389/fmicb.2021.713125 PMC8437397 34526977

[21] Motamedi, E. et al. Efficient removal of various textile dyes from wastewater by novel thermo-halotolerant laccase. *Bioresour Technol.* **337**, 125468 (2021). 34320748 10.1016/j.biortech.2021.125468

[22] Ariaeenejad, S. et al. In-silico discovery of bifunctional enzymes with enhanced lignocellulose hydrolysis from microbiota big data. *Int. J. Biol. Macromol.* **177**, 211–220 (2021). 33549667 10.1016/j.ijbiomac.2021.02.014

[23] Ariaeenejad, S., Motamedi, E. & Hosseini, G. Application of the immobilized enzyme on magnetic graphene oxide nano-carrier as a versatile bi-functional tool for efficient removal of dye from water. *Bioresour Technol.* **319**, 124228 (2021). 33254455 10.1016/j.biortech.2020.124228

[24] Thakrar, F. J. & Singh, S. P. Catalytic, thermodynamic and structural properties of an immobilized and highly thermostable alkaline protease from a haloalkaliphilic actinobacteria, Nocardiopsis alba TATA-5. *Bioresour Technol.* **278**, 150–158 (2019). 30685619 10.1016/j.biortech.2019.01.058

[25] Mehdi, W. A., Mehde, A. A., Özacar, M. & Özacar, Z. Characterization and immobilization of protease and lipase on chitin-starch material as a novel matrix. *Int. J. Biol. Macromol.* **117**, 947–958 (2018). 29807075 10.1016/j.ijbiomac.2018.04.195

[26] Ranjbari, N. et al. Improved features of a highly stable protease from Penaeus vannamei by immobilization on glutaraldehyde activated graphene oxide nanosheets. *Int. J. Biol. Macromol.* **130**, 564–572 (2019). 30831167 10.1016/j.ijbiomac.2019.02.163

[27] Murugappan, G., Khambhaty, Y. & Sreeram, K. J. Protease immobilized nanoparticles: a cleaner and sustainable approach to dehairing of skin. *Appl. Nanosci.* **10**, 213–221 (2020).

[28] Holyavka, M. et al. Novel biotechnological formulations of cysteine proteases, immobilized on chitosan. Structure, stability and activity. *Int. J. Biol. Macromol.* **180**, 161–176 (2021). 33676977 10.1016/j.ijbiomac.2021.03.016

[29] Norouzi, S., Hajati, N., Maghami, P. & Ariaeenejad, S. Improvement of PersiXyn2 activity and stability in presence of Trehalose and proline as a natural osmolyte. *Int. J. Biol. Macromol.* **163**, 348–357 (2020). 32629052 10.1016/j.ijbiomac.2020.06.288

[30] Duman, Y. A. & Tekin, N. Kinetic and thermodynamic properties of purified alkaline protease from Bacillus pumilus Y7 and non-covalent immobilization to poly (vinylimidazole)/clay hydrogel. *Eng. Life Sci.* **20**, 36–49 (2020). 32625045 10.1002/elsc.201900119 PMC6999066

[31] Morellon-Sterling, R. et al. Advantages of supports activated with divinyl sulfone in enzyme coimmobilization: possibility of multipoint covalent immobilization of the most stable enzyme and immobilization via ion exchange of the least stable enzyme. *ACS Sustain. Chem. Eng.* **9**, 7508–7518 (2021).

[32] Siar, E. H., Morellon-Sterling, R., Fernandez-Lafuente, R. & Z. & Use of glyoxyl-agarose immobilized ficin extract in milk coagulation: unexpected importance of the ficin loading on the biocatalysts. *Int. J. Biol. Macromol.* **144**, 419–426 (2020). 31857160 10.1016/j.ijbiomac.2019.12.140

[33] Bilal, M., Ahmad, S. & Carballares, D. Fernandez-lafuente, R. Proteases immobilized on nanomaterials for biocatalytic, environmental and biomedical applications: advantages and drawbacks. *Biotechnol. Adv.* **70**, 108304 (2024). 38135131 10.1016/j.biotechadv.2023.108304

[34] Ariaeenejad, S., Kavousi, K., Mamaghani, A. S. A., Ghasemitabesh, R. & Hosseini Salekdeh, G. Simultaneous hydrolysis of various protein-rich industrial wastes by a naturally evolved protease from tannery wastewater microbiota. *Sci. Total Environ.* **815**, 152796 (2022). 34986419 10.1016/j.scitotenv.2021.152796

[35] Zahirinejad, S. et al. Nano-organic supports for enzyme immobilization : Scopes and perspectives. *Colloids Surf. B Biointerfaces.* **204**, 111774 (2021). 33932893 10.1016/j.colsurfb.2021.111774

[36] Maeda, T., Yoshimura, T., García-Contreras, R. & Ogawa, H. I. Purification and characterization of a serine protease secreted by Brevibacillus sp. KH3 for reducing waste activated sludge and biofilm formation. *Bioresour Technol.* **102**, 10650–10656 (2011). 21925876 10.1016/j.biortech.2011.08.098

[37] Shakeri, F., Ariaeenejad, S., Ghollasi, M. & Motamedi, E. Synthesis of two novel bio–based hydrogels using sodium alginate and chitosan and their proficiency in physical immobilization of enzymes. *Sci. Rep* **12**, 2072 (2022). 35136126 10.1038/s41598-022-06013-0 PMC8827098

[38] Bradford, N. A rapid and sensitive method for the quantitation microgram quantities of a protein isolated from red cell membranes. *Anal. Biochem.* **72**, e254 (1976). 10.1016/0003-2697(76)90527-3 942051

[39] Uane, L. et al. Production of cellulose nanocrystals from pineapple crown fibers through alkaline pretreatment and acid hydrolysis under different conditions. *J. Mater. Res. Technol.* **9**, 12346–12353 (2020).

[40] Zhou, L. et al. One-pot Preparation of Carboxylated Cellulose Nanocrystals and their Liquid Crystalline behaviors. *ACS Sustain. Chem. Eng.* **6**, 12403–12410 (2018).

[41] Liu, S. et al. Smart chemistry of enzyme immobilization using various support matrices –A review. *Int. J. Biol. Macromol.* **190**, 396–408 (2021). 34506857 10.1016/j.ijbiomac.2021.09.006

[42] Shih, Y. et al. Trypsin-immobilized metal–Organic Framework as a Biocatalyst in Proteomics Analysis. *Chempluschem*. **77**, 982–986 (2012).

[43] Dai, X. et al. Preparation, characterization and catalytic behavior of pectinase covalently immobilized onto sodium alginate/graphene oxide composite beads. *Food Chem.* **253**, 185–193 (2018). 29502820 10.1016/j.foodchem.2018.01.157

[44] Zhu, L., Kumar, V. & Banker, G. S. Examination of oxidized cellulose as a macromolecular prodrug carrier: preparation and characterization of an oxidized cellulose-phenylpropanolamine conjugate. *Int. J. Pharm.* **223**, 35–47 (2001). 11451630 10.1016/s0378-5173(01)00725-6

[45] Bouhaine, N., Djerourou, A., Innocent, C. & Seta, P. Preparation of Catalytic materials by trypsin immobilisation on Carboxylic Textile and Carbon matrixes activated by Dicyclohexylcarbodiimide (DCC). *Mater. Sci. Indian J.* **3**, 61–68 (2006).

[46] Montanari, S., Roumani, M., Heux, L. & Vignon, M. R. Topochemistry of Carboxylated Cellulose nanocrystals resulting from TEMPO-Mediated oxidation. *Macromolecules* **38**, 1665–1671 (2005).

[47] Strand, A., Sundberg, A., Arstila, K. & Retulainen, E. Cellulose nano fibrils prepared by gentle drying methods reveal the limits of Helium ion microscopy imaging. *RSC Adv.* **9**, 15668–15677 (2019). 35514833 10.1039/c9ra01447k PMC9064282

[48] Monajati, M., Borandeh, S., Hesami, A., Mansouri, D. & Tamaddon, A. M. Immobilization of L-asparaginase on aspartic acid functionalized graphene oxide nanosheet: enzyme kinetics and stability studies. *Chem. Eng. J.* **354**, 1153–1163 (2018).

[49] Ariaeenejad, S., Kavousi, K., Han, J. L., Ding, X. Z. & Salekdeh, G. H. Efficiency of an alkaline, thermostable, detergent compatible, and organic solvent tolerant lipase with hydrolytic potential in biotreatment of wastewater. *Sci. Total Environ.* **866**, 161066 (2023). 36565882 10.1016/j.scitotenv.2022.161066

[50] Ibrahim, A. S. S. et al. Enhancement of alkaline protease activity and stability via covalent immobilization onto hollow core-mesoporous shell silica nanospheres. *Int. J. Mol. Sci.* **17**, 184 (2016). 10.3390/ijms17020184 PMC4783918 26840303

[51] Qamar, S. A., Asgher, M. & Bilal, M. Immobilization of alkaline protease from Bacillus brevis using Ca-Alginate Entrapment Strategy for Improved Catalytic Stability, Silver Recovery, and Dehairing potentialities. *Catal. Lett.* **150**, 3572–3583 (2020).

[52] Das, R., Mishra, H., Srivastava, A. & Kayastha, A. M. Covalent immobilization of β-amylase onto functionalized molybdenum sulfide nanosheets, its kinetics and stability studies: a gateway to boost enzyme application. *Chem. Eng. J.* **328**, 215–227 (2017).

[53] Moharam, M. E., Gamal-eldeen, A. M., El-sayed, S. T. & Production Immobilization and anti-tumor activity of L-Asparaginase of Bacillus sp R36. *J. Am. Sci.* **6**, 157–165 (2010).

[54] Klibanov, A. M. Enzyme stabilization by immobilization. *Anal. Biochem.* **93**, 1–25 (1979). 35035

[55] de Andrade Silva, T., Keijok, W. J., Guimarães, M. C. C., Cassini, S. T. A. & de Oliveira, J. P. Impact of immobilization strategies on the activity and recyclability of lipases in nanomagnetic supports. *Sci. Rep.* **12**, 6815 (2022). 35474328 10.1038/s41598-022-10721-y PMC9042828

[56] Sinha, R. & Khare, S. K. Immobilization of halophilic Bacillus sp. EMB9 protease on functionalized silica nanoparticles and application in whey protein hydrolysis. *Bioprocess. Biosyst Eng.* **38**, 739–748 (2015). 25385659 10.1007/s00449-014-1314-2

[57] Hudson, S., Magner, E., Cooney, J. & Kieran, B. Methodology for the immobilization of enzymes onto mesoporous materials. *J. Phys. Chem. B*. **109**, 19496–19506 (2005). 16853519 10.1021/jp052102n

[58] Gopinath, S. & Sugunan, S. Leaching studies over immobilized a-amylase. Importance of the nature of enzyme attachment. *React. Kinet Catal. Lett.* **83**, 79–83 (2004).

[59] Azanaw, A., Birlie, B., Teshome, B. & Jemberie, M. Textile effluent treatment methods and eco-friendly resolution of textile wastewater. *Case Stud. Chem. Environ. Eng.* **6**, 100230 (2022).

[60] Remya, R. R. et al. Role of nanoparticles in biodegradation and their importance in environmental and biomedical applications. *J. Nanomater* **2022**, 6090846 (2022).

[61] Girelli, A. M., Astolfi, M. L. & Scuto, F. R. Agro-industrial wastes as potential carriers for enzyme immobilization: a review. *Chemosphere*. **244**, 125368 (2019). 31790990 10.1016/j.chemosphere.2019.125368

[62] Ansari, S. A. & Husain, Q. Potential applications of enzymes immobilized on/in nano materials: a review. *Biotechnol. Adv.* **30**, 512–523 (2012). 21963605 10.1016/j.biotechadv.2011.09.005

